# Investigation of the Relationship between Sagittal Skeletal Nasal Profile Morphology and Malocclusions: A Lateral Cephalometric Film Study

**DOI:** 10.3390/diagnostics13030463

**Published:** 2023-01-27

**Authors:** Yunus Ocak, Orhan Cicek, Nurhat Ozkalayci, Hande Erener

**Affiliations:** 1 Department of Orthodontics, Faculty of Dentistry, Zonguldak Bulent Ecevit University, Zonguldak 67100, Turkey; 2 Department of Healthcare Management, Boyabat Faculty of Economics and Administrative Sciences, Sinop University, Sinop 57000, Turkey; 3 Department of Orthodontics, Faculty of Dentistry, Tekirdag Namık Kemal University, Tekirdag 59030, Turkey

**Keywords:** nasal profile, morphology, malocclusion, skeletal, growth and development, lateral cephalometric, orthodontics

## Abstract

The aim of this study was to evaluate the relationship between skeletal sagittal nasal profile morphology and sagittal skeletal malocclusions. Regarding lateral cephalometric films, the study was conducted in a total of 135 individuals without any prior orthodontic treatment (mean age of 17.91 ± 1.91), including 49 males (mean age 17.91 ± 1.16) and 86 females (mean age 17.78 ± 1.91 years). The groups were divided into two groups as male and female according to gender, and three groups as skeletal Class 1, Class 2, and Class 3 according to the Steiner’s ANB angle. In addition, skeletal groups were compared within groups by dividing into two groups of male and female. A total of eight parameters, three skeletal sagittal angular (SNA, SNB, and ANB angles), four nasal linear (R-A, N-A, N-ANS, and N-R distances) and one nasal angular (N1-N2/N2-R angle), were measured on each cephalometric film. The arithmetic mean and standard deviation of all measured nasal parameters were calculated. For statistical analysis, independent sample t-test and one-way analysis of variance (One-Way ANOVA) were used for normally distributed data, and Mann Whitney U and Kruskal Wallis tests were used for data that did not show normal distribution. For statistical analysis, *p* < 0.05 was considered significant. R-A, N-A, and N-ANS linear nasal parameters differed significantly between the male and female groups, which were evaluated regardless of the skeletal groups, with a higher rate in males (*p* < 0.05). N-R linear nasal parameter showed a statistically significant difference between skeletal malocclusion groups, which were evaluated regardless of gender. N-R distance was found to be significantly longer in skeletal Class 3 individuals than in Class 1 and 2 individuals (*p* < 0.05). There was no statistically significant difference in nasal bone concavity angle in all groups (*p* > 0.05). R-A and N-A linear nasal parameters showed statistically significant differences between male and female sex groups in all skeletal malocclusion classes (*p* < 0.05). At first, results showed that males had longer measurements than females in all linear nasal parameters. Second, longer measurements were found in all linear nasal parameters in skeletal Class 3 individuals than those in skeletal Class 1 and Class 2 individuals. Third, the nasal bone concavity angle was greater in skeletal Class 2 individuals than the others.

## 1. Introduction

Facial beauty or facial attractiveness is a function of the harmonious balance between all parts of the face, such as the forehead, eyes, nose, and lips. Distortions in the proportions that occur between parts constitute deformed faces [1]. These deformations can be changed with dentofacial orthopedics [2,3], orthognathic surgery [4], and aesthetic hard or soft tissue surgery [5,6,7], alone or in various combinations.

Among all the facial parts, the nose is the most notable of the facial structures. It is an organ that contributes to the person’s unique facial characteristics, along with the lips and chin, due to its location in the center of face [8,9]. The increase in minor rhinoplasty applications in recent years supports the prominence of the nose in aesthetic perception. Cankaya et al. [10] examined the effect of different nose types on facial aesthetics, and stated that having a nose with a convex profile in society creates more aesthetic problems than having a retrognathic profile. Thus, these conditions lead orthodontists and plastic surgeons, who have an increase in facial aesthetics as one of their main treatment goals, to research further the relationships between the parts of the face, with increasing interest [1].

The effect of the nose, which is one of the main elements of facial aesthetics, on the face after orthodontic treatment can change positively or negatively, especially after orthognathic surgery. The nasal projection is affected by the forward and backward movements of the lower and upper jaws and teeth, causing it to be one of the structures that undergoes the most changes [11]. Thus, an orthodontist should have a comprehensive knowledge and understanding of the soft tissue changes that may occur during treatment, and the growth and age-related changes in nasal morphology [12,13].

The skeletal nasal aperture, a pear-shaped opening, is a structure formed by the nasal bones at the top, the frontal process of the maxilla on both side walls, and the palatal processes at the base [14,15]. The nasal bone and piriformis aperture are the two main skeletal factors that contribute to the formation of the nose itself, its original shape, and facial structures. The morphological features of the nasal bone and piriformis aperture are widely used for surgical and aesthetic purposes in nasal reconstruction and rhinoplasty, for gender determination in anthropology, and for reconstructing the face of dead individuals in forensic cases [16].

The nasal bone growth is completed by the age of 10. After that time, further growth of the nasal bone depends only on nasal cartilage and soft tissues [17]. On the other hand, the maxillary bone growth, substantially, is completed by the age of 16 [18].

The discovery of X-rays by Roentgen in 1895 revolutionized dentistry, and allowed the radiographic image of the head to be evaluated in two dimensions. Thus, it enabled the proper examination of the growth and development of the head and face [19]. In 1931, standardizable lateral cephalometric X-ray films started to be obtained with the head fixation appliance, called a cephalostat, by Broadbent [20]. In the later years, with the acquisition of standardized cephalometric films, many researchers developed various cephalometric film analysis methods for their own orthodontic treatment planning which are named after them [21,22,23,24,25].

In modern orthodontics, the primary diagnostic method used in the evaluation of skeletal tissues is cephalometric analysis. Cephalometric analyses are often used to determine the positions of the upper and lower jaws [26].

When we research the literature, there are studies that investigate the morphometry and profile of the nose, the changes that occur with orthopedic, orthodontic, and orthognathic treatments, and how it is shaped by growth and development. However, studies examining the relationship between the structures that make up the skeletal structure of the nose and malocclusions are limited.

The aim of this study was to investigate whether there was a relationship between linear and angular measurements, consisting of the points forming the skeletal nasal profile and sagittal skeletal malocclusions formed according to the ANB angle, in individuals whose growth and development have finished, and who have not received prior orthodontic treatment. In addition, it was evaluated whether there was a difference between the genders regarding the skeletal nasal profile. 

## 2. Materials and Methods 

### 2.1. Ethic and Sample Selection

The ethical approval was obtained from the Non-Invasive Clinical Research Ethics Committee of Zonguldak Bulent Ecevit University (No: 2020/22-15-18/11/2020). The material of our study consists of archive records of lateral cephalometric radiographs taken before starting the treatment in a total of 135 individuals, 49 males and 86 females, who were referred to Zonguldak Bulent Ecevit University, Department of Orthodontics. The age range of the individuals included in the study was 16–40 years, with a mean age of 17.88 ± 3.14 years.

The criteria for inclusion in the study are presented below.

Inclusion criteria:

- Individuals over 16 years of age;
- Being in the post pubertal period (Ru according to the hand-wrist radiography or CS5 or CS6 according to the cervical vertebral maturation classification) that has passed the growth spurt stage [27,28];
- No prior orthodontic treatment;
- No tooth loss;
- Not having undergone any nasal surgery or aesthetic operation;
- Not having a facial pathology or/and syndrome;
- No nasal trauma.

### 2.2. Cephalometric Analysis

In the study, Steiner’s cephalometric analysis was used to determine the skeletal facial patterns of individuals by using Nemoceph NX 2006 (Madrid, Spain) cephalometric analysis program. A total of 8 parameters, 3 sagittal skeletal angular and 5 nasal skeletal (4 linear and 1 angular), were measured on standardized lateral cephalometric films (Table 1).

### 2.3. Sample Groups

Individuals included in the study were divided into 2 groups, female and male, to evaluate the gender differences in nasal parameters, regardless of their skeletal malocclusions. Then, to determine the difference between skeletal malocclusions in all individuals included in the study, regardless of gender, skeletal Class 1 (4° ≥ ANB ≥ 0°), skeletal Class 2 (ANB > 4°), and skeletal Class 3 (ANB ˂ 0°) according to the ANB angle of Steiner cephalometric film analysis divided the participants into 3 groups [29]. In addition, each skeletal group was evaluated as male and female within the group.

**Figure 1:**
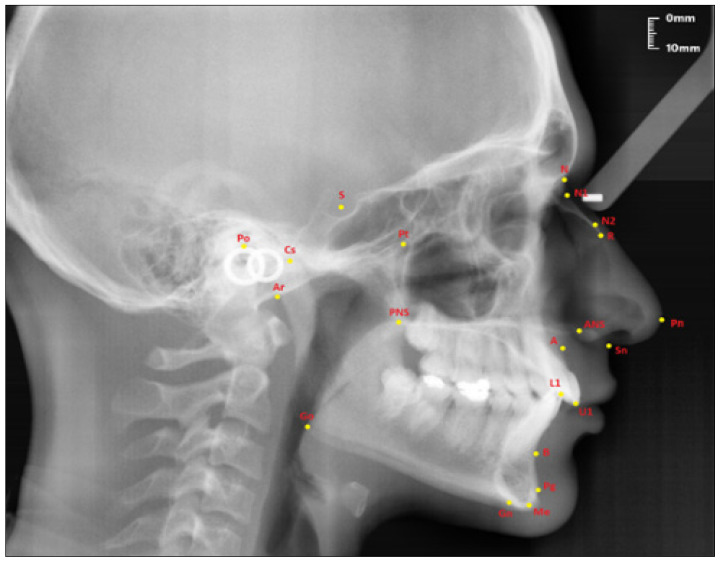
Points used in the study.

The points used in lateral cephalometric films to evaluate the nasal structure are as follows [1,30]. (see Figure 1)

1. Sella (S); the center of Sella turcica.
2. Nasion (N); the most anterior point of the sutura frontonasalis and the deepest place of the recess in that region.
3. Rhinion (R); the most anterior and inferior point on the tip of the nasal bone.
4. Subspinal–point A; below the ANS point, the maxilla is the deepest point of the alveolar bone recess.
5. Supramental–point B; deepest midline point on the mandible between infradentale and pogonion.
6. Anterior nasal spine (ANS) point; the tip of the median, sharp bony process of the maxilla at the lower margin of the anterior nasal opening.
7. N1 point; the most concave point of the nasal bone.
8. N2 point; the most convex point of the nasal bone.

The following linear and angular measurements in relation to the nasal bone were used to evaluate the nose [1,31].

**Figure 2:**
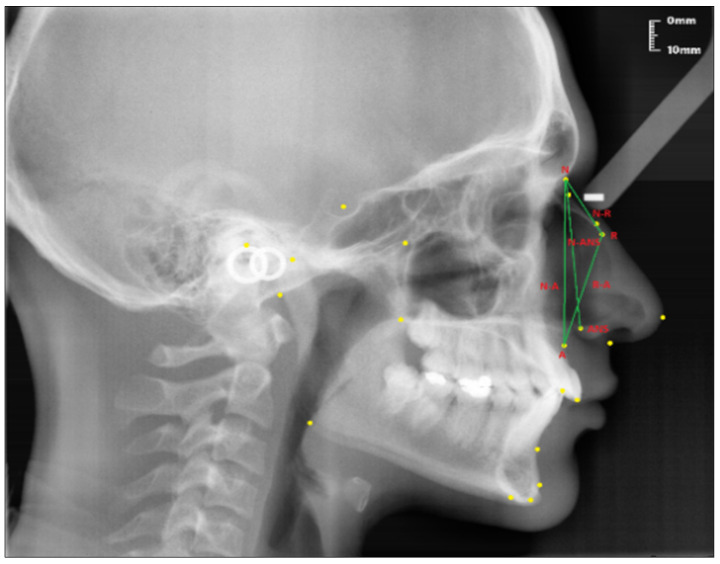
Linear measurements of the nasal profile used in the study.

Linear measurements (see Figure 2):

1. Nasion-Rhinion (N-R); the distance between the nasion and the rhinion points.
2. Nasion-Anterior nasal spina (N-ANS); the distance between the nasion and the anterior nasal spina points.
3. Rhinion-Subspinale (R-A); the distance between the rhinion and the subspinale points.
4. Nasion-Subspinale (N-A); the distance between the nasion and the subspinale points.

**Figure 3:**
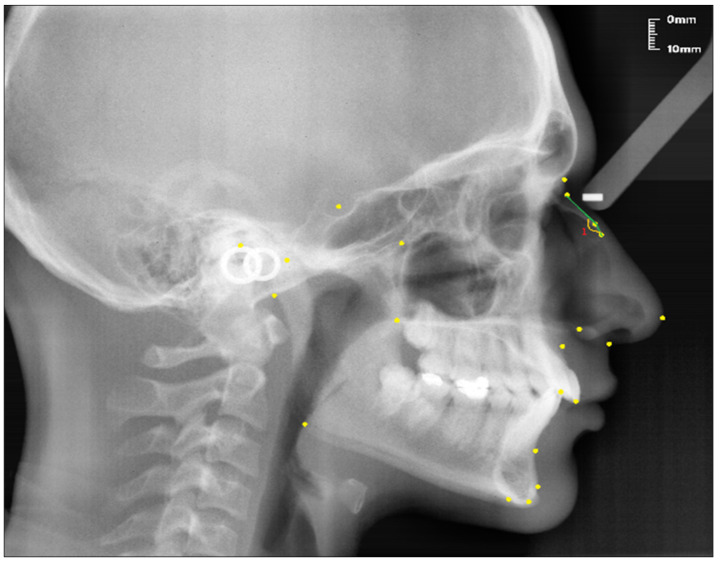
Angular measurement of the nasal bone used in the study.

Angular measurement (see Figure 3):

1. Nasal Bone Concavity Angle (Nbone Angle); the posterior angle formed between the N1-N2 line and the N2-R line.

**Figure 4:**
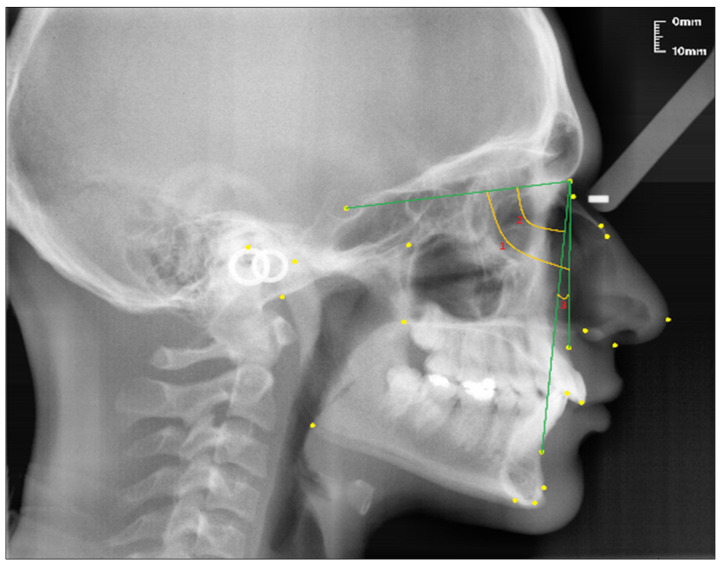
Angular craniofacial skeletal measurements used in the study.

The following parameters were used to classify skeletal malocclusions [29,30] (see Figure 4).

1. SNA (°); the angle between the SN line and the NA line.
2. SNB (°); the angle between the SN line and the NB line.
3. ANB (°); the angle between the lines NA and NB.

### 2.4. Statistical Analysis

Data obtained from lateral cephalometric films were analyzed using the IBM SPSS statistical analysis program (version 25, SPSS, IBM Corporation, New York, NY, USA). Standard deviations, means, minimum, and maximum values of each parameter were calculated. Before evaluating the differences between the groups, a normality test was applied to the data, independent sample t-test and One-Way ANOVA were used in case of normality assumptions, and non-parametric Kruskal Wallis and Mann Whitney U tests, which are the equivalent of parametric tests, were used in cases that did not provide normal distribution. The Shapiro–Wilk test was used to examine the conformity of the data to the normal distribution. *p* < 0.05 was considered to be statistically significant.

## 3. Results

According to the skeletal sagittal classification findings with Steiner cephalometric analysis, the number of 53, 56, and 26 individuals were found to be skeletal Class 1, Class 2, and Class 3, respectively. Gender distribution for Class 1 was 20 males and 33 females; for Class 2 was 17 males and 39 females; and for Class 3 was 12 males and 14 females. Data on skeletal sagittal classifications were given in Table 2.

When the nasal parameters were examined independently of skeletal malocclusions between male and female groups, statistically significant differences were found between male and female R-A, N-A, and N-ANS nasal parameters (*p* < 0.05). It was observed that males had significantly longer linear nasal parameter measurements than females in all three parameters (Table 3).

When the nasal parameters were evaluated according to the sagittal skeletal malocclusion classification, only the N-R parameter showed a statistically significant difference (*p* < 0.05). The N-R parameter was measured as the longest in skeletal Class 3 individuals, and the shortest in skeletal Class 1 individuals. In addition, no statistically significant difference was observed between the skeletal Class 1, 2, and 3 groups in nasal parameters R-A, N-A, and N-ANS (*p* > 0.05) (Table 3).

In the pairwise comparison of N-R nasal parameters between skeletal Class 1, Class 2, and Class 3 malocclusions, skeletal Class 3 individuals showed statistically significant differences from skeletal Class 1 and Class 2 individuals (*p* < 0.05). Skeletal Class 3 individuals showed longer distance values than other skeletal classifications. In the pairwise comparison of other skeletal classifications, it was found that there was no statistically significant difference in the N-R distance (*p* > 0.05) (Table 4). 

Nasal bone concavity angle did not show a statistically significant difference between males and females (*p* > 0.05) (Table 5).

There was no statistically significant difference between nasal bone concavity angle and skeletal Class 1, Class 2, and Class 3 individuals (*p* > 0.05) (Table 5).

Nasal bone concavity angle was seen as highest in skeletal Class 2 and lowest in skeletal Class 3 individuals (Table 5).

R-A and N-A nasal linear parameters showed statistically significant differences between male and female groups in all skeletal groups (*p* < 0.05). R-A and N-A nasal parameters were found to be longer in males than in females (*p* < 0.05) (Table 6). 

N-R, N-ANS, and N1-N2/N2-R values did not differ significantly between genders depending on skeletal groups (*p* > 0.05) (Table 6).

## 4. Discussion

There are many methods for measurements that can be made on the nose. In addition to the use of cephalometric films, standardized photographs, cone-beam computed tomography (CBCT), stereophotogrammetries, clinical measurements taken directly from the patient, and nasolabial models can also be used in studies [32,33,34,35,36,37]. Although the use of 3D data is increasing nowadays to eliminate some of the disadvantages of two-dimensional cephalometric analyses, greater X-ray exposure compared to cephalometric films, the inadequacy of tools in the analysis of patient records, and CBCT data are still the main limitations in their widespread use today [38].

In the present study, lateral cephalometric films were used in the evaluation of nasal parameters because lateral cephalometric films are routinely taken for diagnosis and treatment planning, they do not have any additional cost, they do not have any side effects, they can be obtained in a practical way for the patient and the physician, their standardization can be carried out, and the measurements are reliable.

Increasing facial aesthetics is one of the main goals of orthodontic treatment, and has an important effect on facial aesthetics because the nose is located in the center of the face [39]. Thus, the change in its form, shape, dimensions, and proportions has made the nose an important component that affects the facial profile and adds character to the face. This makes it necessary for the orthodontist to include the nose in diagnosis and treatment planning, especially considering the potential for future growth and changes in the shape of the nose in their profile analysis [12,40,41]. From the orthodontic perspective, the nose is a structure whose relationship with other facial elements is evaluated to provide ideal aesthetics in treatment planning, especially in camouflage and orthognathic treatments [42].

In previous studies, it was seen that studies investigating the relationship between nasal morphology and orthodontic skeletal malocclusions were mostly conducted on the soft tissues of the nose. In the literature, studies examining the relationship between the nasal skeletal profile and skeletal malocclusions were limited. This study will shed light on the literature, in terms of examining the relationship between the skeletal nasal profile and both gender and sagittal skeletal malocclusions. The nasal bone, which forms the upper border of the nasal skeleton, completes its growth around the age of 10, and the maxilla, which forms the lateral wall, completes most of its growth at the age of 16 in both genders. Therefore, individuals aged 16 and over were included in this study.

Karadağ et al. [43] conducted a study in the Anatolian Turkish population and found that nasal bone length was 30.61 ± 1.26 mm in males and 29.01 ± 1.12 mm in females. Hwang et al. [44] reported that nasal bone length was 25.9 ± 3.8 mm in males and 24.5 ± 3.7 mm in females in their study conducted in a Korean population. Lang and Baumeister [45] evaluated nasal bone length in the German population without gender discrimination and found it to be 24.9 ± 3.2 mm. In a similar study, Ofodile [46] stated the nasal bone length as 3.02 cm in Austrians and 2.79 cm in black Americans. Gülşen et al. [1], in their study examining the relationship between facial skeletal structures and nasal profile in Anatolian Turkish adults, stated that nasal bone length was not associated with ANB angle, that is, with skeletal sagittal malocclusions and gender. However, they stated that a long nasal bone can be expected especially in individuals with a long nose, posteriorly positioned and vertically long maxilla, and increased anterior and posterior facial heights.

In the present study, nasal bone length was found to be 24.96 ± 3.73 mm in males and 24.32 ± 3.12 mm in females. Although there was no statistical significance in terms of nasal bone lengths between both genders, the fact that nasal bone lengths were longer in males than in females is consistent with other studies in the literature. According to skeletal sagittal malocclusions, regardless of gender, nasal bone length was found to be 24.13 ± 3.11 mm in skeletal Class 1 individuals, 24.22 ± 3.42 mm in skeletal Class 2 individuals, and 26.12 ± 3.36 mm in skeletal Class 3 individuals. McNamara [47] stated that adults with skeletal Class 3 malocclusion have a 65–67% retrusive maxilla, and often an increased lower face height. Therefore, Gülşen et al. [1] thought that the reason why the nasal bone length in skeletal Class 3 individuals was longer than other skeletal sagittal malocclusions may be due to the increase in lower anterior face height and retrusive maxilla in skeletal Class 3 individuals.

Başçiftçi et al. [48] conducted a study on Class 1 occlusion, normal growth-development, and good facial symmetry including 110 young adult Turkish individuals (55 females, 55 males). They found the distance between N and ANS as 56.82 ± 5.87 mm on average in the whole population, and 58.80 ± 7.15 mm in males and 54.83 ± 3.28 mm in females. Lighthelm et al. [49] stated that they found the N-ANS distance to be 56.1 ± 3.8 mm in 22-year-old adult males and 50.5 ± 2.7 mm in females in 60 normal individuals selected from the Nijmegen Growth Study. Genecov et al. [50] determined the skeletal N-ANS distance of 17-year-olds as being 54.6 ± 3.2 mm in Class 1 males, 56.4 ± 3.2 mm in Class 2 males, 53.2 ± 2.2 mm in Class 1 females, and 51.9 ± 2.4 mm in Class 2 females.

In our study, the skeletal N-ANS distance was found to be 54.32 ± 3.45 mm in males and 51.81 ± 3.05 mm in females, regardless of sagittal skeletal classification. Upper anterior face height differs between male and female individuals. In the studies in the literature, it is seen that the height of the upper anterior face differs between male and female individuals, and males have a higher anterior face height than females. The presented study is consistent with the literature. Regardless of gender, skeletal Class 1, skeletal Class 2, and skeletal Class 3 individuals were 51.96 ± 3.07 mm, 52.97 ± 3.50 mm, 53.72 ± 3.41 mm, respectively. Ardani et al. [51] stated that N-ANS distance in Class 2 individuals is related to the SNB angle. It has been reported that the decrease in the SNB angle will show the back-down rotation of the mandible, and that the upper anterior face height will increase together with the total anterior face height. Thus, the reason why N-ANS distance is longer in skeletal Class 2 individuals than in skeletal Class 1 individuals in the study may be due to the increased facial height in skeletal Class 2 individuals due to posterior rotation of the mandible. Bhushan et al. [52] evaluated the relationship between maxillary rotation and nasal morphology in 45 male individuals aged between 18 and 30 years, and stated a positive correlation with the clockwise rotation of the maxilla. In our study, it was thought the reason that the N-ANS distance in skeletal Class 3 individuals was longer than other skeletal malocclusions might be due to the retrognathic maxilla of skeletal Class 3 individuals and, accordingly, the clockwise rotation of the maxilla.

Arshad et al. [53] evaluated the nasal bone concavity angle, independently of sagittal skeletal malocclusions, in the Pakistani population, and found it to be 167.16° ± 9.07° in males and 166.37° ± 11.21° in females. Gülşen et al. [1], in their study on the Turkish population, found that nasal bone concavity angle was significant when skeletal sagittal and vertical malocclusions were evaluated together. According to their study, they stated that the nasal bone concavity angle was 165° ± 8.36° in skeletal Class 1 low-angle (short face) individuals, 170° ± 7° in normo-angle individuals, and 171° ± 6.93° in high-angle (long face) individuals.

In the present study, the nasal bone concavity angle was found to be 162.11° ± 6.89° in males and 163.60° ± 6.30° in females. In addition, the nasal bone concavity angle was found to be 162.11° ± 6.89° in males and 163.60° ± 6.30° in females. When evaluated according to skeletal malocclusions, regardless of gender, it was found to be 162.45° ± 7.16° in skeletal Class 1 individuals, 164.25° ± 6.28° in skeletal Class 2 individuals, and 161.71° ± 5.52° in skeletal Class 3 individuals. When compared with the present study, nasal bone concavity angle was higher in both gender groups in Pakistani individuals. It was thought that this might be due to ethnic differences. In addition, vertical malocclusions were grouped in the study of Gülşen et al. [1]. Therefore, the evaluated ethnicity may be the reason why the values are different from the presented study, even though it is the same ethnicity. Although the nasal bone length is longer in skeletal Class 3 individuals, the lower nasal bone concavity angle is thought to be because the maxilla is located in a more backward position compared to other skeletal patterns.

A study investigating the relationship between R-A and N-A parameters and skeletal malocclusions has not been found in the literature. In the present study, it was found that there was no statistical significance between skeletal malocclusions in both parameters. However, in both parameters, there was a significant difference between the genders, with males having longer measurements. The R-A parameter was found to be 42.59 ± 3.05 mm in males and 38.60 ± 2.87 mm in females. The N-A parameter was found to be 59.35 ± 3.98 mm in males and 55.95 ± 3.52 mm in females.

The timing of puberty makes a significant difference to final body size. Those who enter puberty early are shorter and those who enter late are taller. Premature termination of growth due to early sexual maturation is particularly evident in females. This is the biggest reason for the size difference between adult males and females [17]. Therefore, just as males in the N-R and N ANS linear parameters show longer values, it is expected that the R-A and N-A linear parameters will show higher values in males, and this was also found in our study.

The development of the face, jaw and teeth, including the nose, is basically the result of a multi-layered and complex interaction of factors that we can call genetic and environmental factors [54]. The strong influence of hereditary inheritance on facial features is manifested by familial predispositions in nasal curvature, chin shape, and smile. One of the best examples of heredity on the jaw is in the Austrian Habsburg Royal Family [17,55]. Today, heritability is known not only for mandibular prognathism, but also for other types of malocclusion such as open bite and class 2 division 2 [56,57,58]. Environmental factors act on the face and its constituent components through the pressures and forces associated with physiological activity during growth and development [17]. Again, environmental factors take part in growth and development processes by enabling the activation and inactivation of relevant gene regions, without making any changes in the DNA sequence. This condition is known as epigenetics. In this way, the determination of facial growth and shaping by both genetic material and environmental factors, that is, the ‘complex trait (genetic and environmental factors acting together and jointly)’, makes predictability difficult. It leads to the formation of millions or even billions of combinations [59]. Therefore, as in the present study, it is very difficult to fully explain the relationship between the nose and malocclusions with only millimetric or angular measurements. It is thought that artificial intelligence software can be useful to reveal a predictable relationship by creating these combinations. The rate of variability in heritable characteristics can be estimated by comparing identical twins, fraternal twins, and ordinary siblings. The classic way to determine how much of a characteristic is inherited is to compare it with monozygotic and dizygotic twins. Thus, the hereditary and environmental components of a trait can be distinguished [17]. The individuals included in our study do not consist of twin individuals, that is, they differ genetically. Even if the individuals participating in the study are twins, it is not known which environmental conditions they are under, and how long they have been exposed to these conditions. This study is limited in this aspect.

## 5. Conclusions

The obtained conclusions within the limits of our study are as follows:

1. All nasal dimensions were longer in males than females;
2. It was observed that skeletal Class 3 individuals had longer nasal linear parameters than skeletal Class 1 and skeletal Class 2 individuals;
3. Although the nasal bone concavity angle was greater in skeletal Class 2 individuals, it was not associated with other malocclusions;
4. For the sake of detailed and distinctive findings, there is a need for new studies to be conducted on identical twins, fraternal twins, and ordinary siblings. In addition, some investigations should be taken to determine the effects of heredity and environment on nasal growth and development, by forming groups in a larger sample in vertical and transversal directions.

## Figures and Tables

**Table 1:** Parameters used in the study.

Parameters		
Skeletal Angular Parameters	Nasal Parameters	
	Linear Parameters	Angular Parameter
SNA SNB ANB	N-R R-A N-A N-ANS	N1N2-N2R (Nasal bone concavity angle)

**Table 2:** Data of the skeletal sagittal classifications according to the gender of the individuals included in the study (n = 135).

	Skeletal Class 1 (n/%)	Skeletal Class 2 (n/%)	Skeletal Class 3 (n/%)
Males	20/14.8	17/12.5	12/9
Females	33/24.4	39/28.8	14/10.5
Total	53/39.2	56/41.3	26/19.5

n; Number of individuals, %; Percent.

**Table 3:** Comparison of nasal parameters between the groups.

	Distance N-R (mm)		Distance R-A (mm)		Distance N-A (mm)		Distance N-ANS (mm)	
	*$\overset{-}{x}$ ± ss*	*p*	*$\overset{-}{x}$ ± ss*	*p*	*$\overset{-}{x}$ ± ss*	*p*	*$\overset{-}{x}$ ± ss*	*p*
Gender ^+^								
Males	24.96 ± 3.73	0.283	42.59 ± 3.05	0.000 *	59.35 ± 3.98	0.000 *	54.32 ± 3.45	0.000 *
Females	24.32 ± 3.12		38.60 ± 2.87		55.95 ± 3.52		51.81 ± 3.05	
Skeletal Classification ^++^								
Class 1	24.13 ± 3.11	0.028 *	39.60 ± 2.92	0.493	56.36 ± 3.39	0.084	51.96 ± 3.07	0.073
Class 2	24.22 ± 3.42		40.28 ± 3.73		57.37 ± 4.10		52.97 ± 3.50	
Class 3	26.12 ± 3.36		40.54 ± 4.08		58.46 ± 4.79		53.72 ± 3.41	

^+^: independent sample *t*-test, ^++^: one-way ANOVA Test, $\overset{-}{x}$: arithmetic mean, *ss*: standard deviation, mm: millimeter, *p*: significance level, *: *p* < 0.05.

**Table 4:** Comparison of N-R distance between the skeletal malocclusions.

Skeletal Classification	Skeletal Classification	Average Difference	*p*
Class 1	Class 2	−0.09068 ± 0.63	0.989
	Class 3	−1.99373 ± 0.78	0.034 *
Class 2	Class 1	0.09068 ± 0.63	0.989
	Class 3	−1.90305 ± 0.78	0.043 *
Class 3	Class 1	1.99373 ± 0.78	0.034 *
	Class 2	1.90305 ± 0.78	0.043 *

Post-hoc (Tukey test) multiple comparison analysis, *p*: significance level, *: *p* < 0.05.

**Table 5:** Comparison of nasal bone concavity angle with skeletal malocclusions and gender.

N1-N2/N2-R Angle (Nasal Bone Concavity Angle)		
	*$\overset{-}{x}$ ± ss*	*p*
Gender ^+^		
Males	162.11° ± 6.89°	0.206
Females	163.60° ± 6.30°	
Skeletal Classification ^++^		
Class 1	162.45°± 7.16°	0.180
Class 2	164.25° ± 6.28°	
Class 3	161.71° ± 5.42°	

^+^: independent sample *t*-test, ^++^: one-way ANOVA Test, $\overset{-}{x}$: arithmetic mean, *ss*: standard deviation, *p*: significance level.

**Table 6:** Comparison of the nasal-related parameters between genders within the skeletal groups.

Linear Nasal Parameters	Skeletal Classification	Males	Females	
		*$\overset{-}{x}$ ± ss*	*$\overset{-}{x}$ ± ss*	*p*
N-R	Class 1	24.63 ± 3.96	23.83 ± 2.48	0.367
	Class 2	24.67 ± 3.,43	24.02 ± 3.44	0.517
	Class 3	25.93 ± 3.92	26.29 ± 2.99	0.794
R-A	Class 1	41.86 ± 2.20	38.23 ± 2.42	0.000 *
	Class 2	42.76 ± 3.88	39.20 ± 3.14	0.001 *
	Class 3	43.54 ± 2.91	37.80 ± 2.91	0.000 *
N-A	Class 1	58.20 ± 3.88	55.24 ± 2.50	0.001 *
	Class 2	59.63 ± 3.97	56.39 ± 3.80	0.006 *
	Class 3	60.87 ± 3.91	56.39 ± 4.61	0.014 *
N-ANS	Class 1	53.29 ± 3.41	51.16 ± 2.47	0.111
	Class 2	55.13 ± 3.01	52.03 ± 3.31	0.102
	Class 3	54.88 ± 3.93	52.76 ± 3.39	0.153
N1-N2/N2-R	Class 1	162.13 ± 7.90	162.64 ± 6.80	0.803
	Class 2	164.26 ± 6.50	164.25 ± 6.28	0.997
	Class 3	159.03 ± 4.55	164.01 ± 5.16	0.116

Independent sample *t*-test analysis, $\overset{-}{x}$: arithmetic mean, *ss*: standard deviation, *p*: significance level, *: *p* < 0.05.

## Data Availability

The data of this study are not publicly available as they were formed by examining the archive records, and will be used in another study. In addition, all data supporting the results of this study are included within the article.
